# Comparative Efficacy of Photodynamic Therapy Versus Cryotherapy for Actinic Keratosis: A Systematic Review and Meta‐Analysis of Randomized Controlled Trials

**DOI:** 10.1111/jocd.70749

**Published:** 2026-04-11

**Authors:** Saleh Aldraibi, Maya Alharbi, Kholoud Alzubaidi, Omar Turkistani, Abdulmalik Bin Kassim, Dhai Almutairi, Majid Alhammadi, Jumanah Makhtoum, Fadi Alghamdi

**Affiliations:** ^1^ College of Medicine King Saud bin Abdulaziz University for Health Sciences Jeddah Saudi Arabia; ^2^ Faculty of Medicine King Abdulaziz University Jeddah Saudi Arabia; ^3^ Faculty of Medicine Umm Al Qura University Al Qunfudhah Saudi Arabia; ^4^ Faculty of Medicine Imam Mohammad Ibn Saud Islamic University Riyadh Saudi Arabia; ^5^ King Khalid General Hospital Hafer Al Batin Saudi Arabia; ^6^ Sheikh Khalifa medical city Abu Dhabi UAE; ^7^ Department of Dermatology King Fahad Armed Forces Hospital Jeddah Saudi Arabia

**Keywords:** actinic keratosis, aminolevulinic acid hydrochloride, cryotherapy, methyl aminolevulinate, photodynamic therapy

## Abstract

**Background:**

Photodynamic therapy and cryotherapy are treatment options for actinic keratosis; however, their efficacy and safety remain debated.

**Aims:**

To perform a high‐quality systematic review and meta‐analysis exploring the efficacy and safety of photodynamic therapy and cryotherapy in actinic keratosis.

**Methods:**

A systematic search was performed applying the Preferred Reporting Items for Systematic Reviews and Meta‐Analyses guidelines. We searched PubMed, Web of Science, Cochrane, Science Direct, Ovid, EBSCO, Wiley, and Google Scholar for randomized controlled trials.

**Results:**

A total of seven studies with 1233 patients were identified. PDT and cryotherapy showed similar success in clearing lesions (RR, 1.02; 95% CI, 0.92–1.13; *p* = 0.74). While both treatments performed comparably on the head and face (RR, 1.10; 95% CI, 0.94–1.28; *p* = 0.24), data from one trial suggested cryotherapy might be more effective for lesions on the arms and legs (RR, 0.88; 95% CI, 0.82–0.94; *p* < 0.05). However, more research is needed to confirm this finding. Cosmetic outcomes were significantly better for PDT (74.62% vs. 49.11%: RR, 1.52; 95% CI, 1.4–1.65; *p* < 0.00001) than cryotherapy. Similarly, PDT was superior to cryotherapy in patient satisfaction though the overall difference was not statistically significant (RR, 1.43; 95% CI, 0.91–2.25; *p* = 0.12). PDT was associated with a significantly higher risk of burning sensations and pain (RR, 1.95; 95% CI, 1.27–3.02; *p* = 0.002), whereas cryotherapy more frequently led to vesicles and blisters.

**Conclusion:**

Lesion clearance may depend on location. It is comparable for head and face lesions, while data from one trial suggests cryotherapy may be better for extremity lesions. PDT is associated with a higher occurrence of pain/burning, while cryotherapy leads to more vesicles/blisters. Future research should focus on standardized protocols, including blinded post‐treatment assessments to improve reliability and minimize bias.

## 1 Introduction

Actinic keratosis (AK) is a common skin neoplasm with a global prevalence of approximately 14%, and an incidence of 1.9 per 1000 people annually [1, 2]. It predominantly affects elderly, fair‐skinned males with chronic sun exposure [3, 4]. The clinical significance is its potential progression to squamous cell carcinoma (SCC) as Marks et al. reported a 0.06% annual transformation risk based on over 20 000 lesions, and a 10.2%–20% 10‐year risk in patients with multiple lesions in other studies [5, 6, 7]. Cryotherapy is a commonly available and cost‐effective treatment option that obliterates AK by decreasing skin temperature to −50°C and infrequently requires prior anesthesia [3, 8]. Thai et al. reported a 67.2% complete lesion clearance with cryotherapy, while Goldberg et al. observed a 66.7% complete clearance rate of AK lesions at week 1, and 100% at week 6 [9, 10]. Another treatment modality is photodynamic therapy (PDT) using topical Methyl Aminolevulinate (MAL) or Aminolevulinic Acid (ALA) as photosensitizers, and a light source with adequate energy such as blue light (400 nm) and red light (630 nm) [11]. The efficacy of PDT is demonstrated by two studies by Pariser et al. who found the complete response rate of MAL‐PDT using red light versus placebo to be 86.2%–89% versus 38%–52.2%, *p* = 0.001, respectively [12, 13]. Similarly, Szeimies et al. [14] established that MAL‐PDT was significantly superior to placebo, with a complete response rate of 83.3% versus 28.7% (*p* = 0.001). Despite the established efficacy of PDT and cryotherapy, current systematic reviews have not adequately addressed the adverse events (AEs) and complications associated with these treatment modalities. Moreover, recent trials, including those evaluating daylight photodynamic therapy (DL‐PDT) with MAL versus cryotherapy, were not included in previous reviews. This systematic review and meta‐analysis aim to comprehensively compare PDT and cryotherapy in terms of efficacy, safety, and cosmetic results in the management of AK.

## 2 Materials and Methods

### 2.1 Review of the Literature

We applied the PRISMA (Preferred Reporting Items of Systematic Reviews and Meta‐Analyses) approach to ensure the least level of bias [15]. The study protocol is registered under PROSPERO, ID: CRD42024618838 [16]. We conducted a systematic search of the following databases: (1) PubMed, (2) Web of Science, (3) Cochrane, (4) Science Direct, (5) Ovid, (6) EBSCO, (7) Wiley, and (8) Google Scholar, using the following keywords: (Photodynamic therapy OR PDT OR aminolevulinic acid OR methyl aminolevulinate) AND (Cryotherapy OR cryosurgery) AND (Acute keratosis OR actinic keratosis OR AK). Due to the nature of the study, ethical approval was not required.

### 2.2 Methodology for Selecting Studies

We included studies that met the following criteria: (1) written in English, (2) randomized controlled trials (RCTs) with at least 10 participants and follow‐up data, (3) patients diagnosed with AK, regardless of skin type, age, or gender, (4) patients treated with PDT using topical ALA hydrochloride or MAL, (5) studies directly comparing PDT to cryotherapy, and (6) primary outcomes include lesion clearance, cosmetic outcomes, patient satisfaction, and secondary outcomes, such as AEs.

### 2.3 Process of Screening and Data Extraction

Two independent authors (DA and OT) screened and reviewed studies by title and abstract using the Rayyan search web for systematic reviews [17]. Then, the full text was reviewed by another two independent authors (MH and AK), with differences resolved by a third author (SA). Afterwards, data extraction was performed by 2 authors independently (SA and MA).

### 2.4 Assessment of Quality and Bias Risk

Assessment of quality and bias was done using the Cochrane risk‐of‐bias tool (RoB 2) for randomized trials, with domains including randomization process, intended intervention deviation, missing outcome data, outcome measurement, reported result selection, and overall risk [18]. Two authors (SA and KA) assessed the risk of bias, then another author (SA) verified the results.

### 2.5 Meta‐Analysis of the Included Data

Statistical analyses were performed using Review Manager software (RevMan) version 5.4 for the primary meta‐analyses of lesion clearance (Figure 2) and adverse events (Figure 5). For the subgroup analysis by anatomical location (Figure 3) and the leave‐one‐out sensitivity analysis (Figure 4), the R software (version 4.5.1) with the “meta” and “metafor” packages was used. Overall, all results were analyzed using a random‐effects model and expressed as Risk Ratios (RR) with 95% Confidence Intervals (CI).

## 3 Results

### 3.1 Literature findings

**FIGURE 1:**
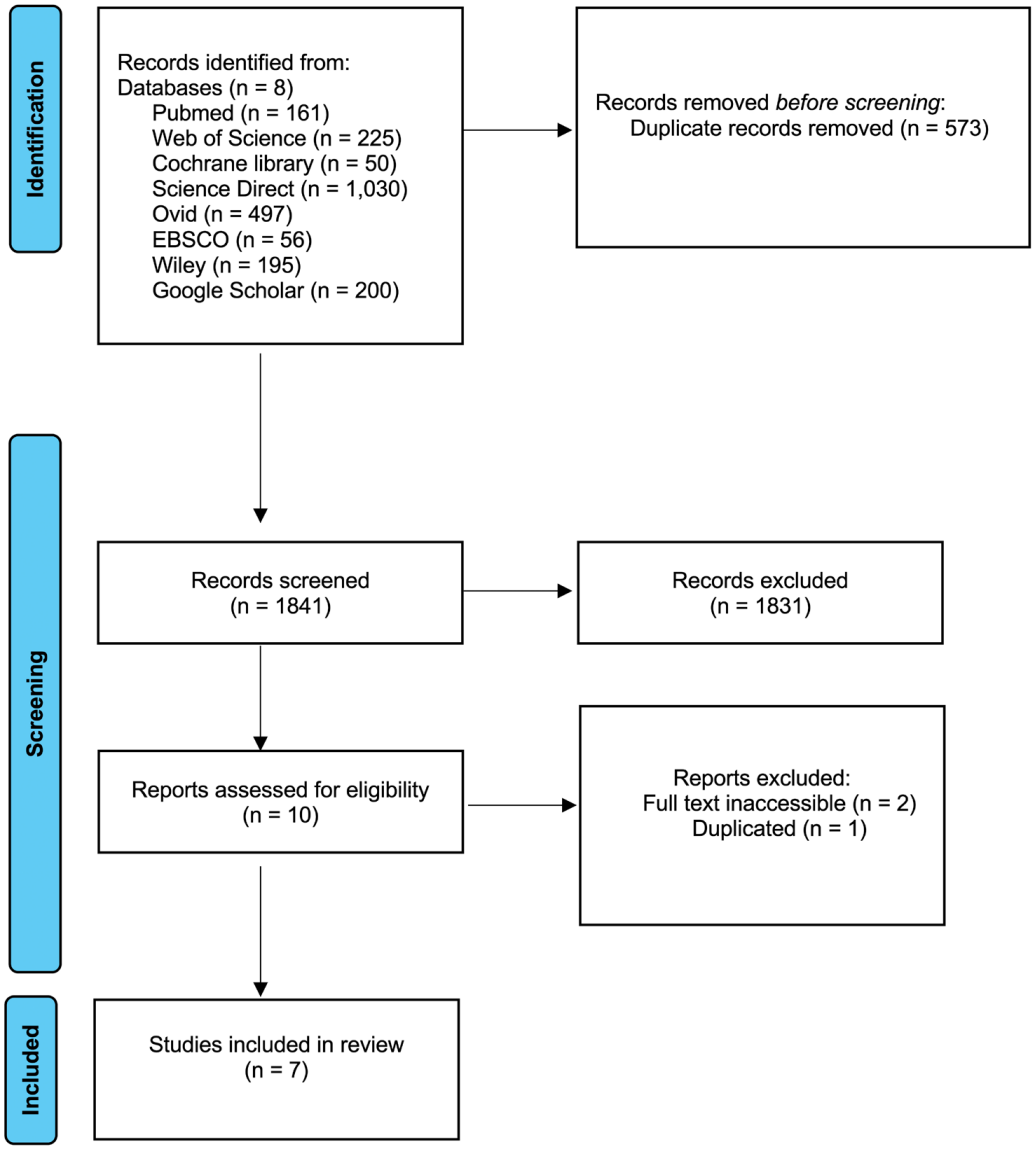
The flow chart of included studies according to PRISMA.

2414 studies were identified through searches in eight databases. After removal of 573 duplicates, 1841 records underwent title and abstract screening. Following full‐text review, 10 studies were assessed for eligibility. After exclusions, 7 studies were included in the qualitative synthesis, and 5 in the meta‐analysis (Figure 1) [19, 20, 21, 22, 23, 24, 25].

### 3.2 Characteristics and Management of the Included Studies

Geographically, three studies conducted in Germany (42.86%) [23, 24, 25], one in the United Kingdom (14.29%) [22], one multicenter study across Europe (14.29%) [20], one in Australia (14.29%) [21], and one multicenter study between Europe and Australia (28.57%) [19]. Demographically, the mean range of age is 64–74.8 years, and there is a male predominance of 68.6% (Table 1). Additionally, five studies utilized topical MAL as a photosensitizer [19, 20, 21, 22, 25], while two studies employed topical ALA [24, 25]. Red light was used in six studies with wavelengths of 570–670 nm, and a light dose between 37 and 76 J/cm^2^. The exposure duration ranged from 8 min and 36 s to 10 min and 56 s. On the other hand, daylight was used as a PDT light source in one study, with a mean exposure duration of 2 h (Table 2).

**TABLE 1:** Characteristics of the included studies.

Article ID	Author, year	Study country	Study design	No. of patients			Age range (years)		Gender, male *N* (%)		Gender, female *N* (%)	
				Total number	Number of patients received PDT	Number of patients received cryotherapy	PDT	Cryotherapy	PDT	Cryotherapy	PDT	Cryotherapy
1	Szeimies et al. 2002 [20]	Europe	RCT	202	102	100	42–88	45–89	66 (64.7)	58 (58.0)	36 (35.3)	42 (42.0)
2	Freeman et al. 2003 [21]	Australia	RCT	204	88	89	33–86	38–86	49 (56)	54 (61)	39 (44)	35 (39)
3	Morton et al. 2006 [22]	United Kingdom	RCT	119	119	119	53.9–93.3	53.9–93.3	108 (90.76)	108 (90.76)	11 (9.24)	11 (9.24)
4	Kaufmann et al. 2008 [19]	Europe& Australia	RCT	121	121	121	NA	NA	78 (64.5)	78 (64.5)	43 (35.5)	43 (35.5)
5	Hauschild et al. 2009 [23]	Germany	RCT	349	148	149	41–94	41–93	104 (70)	104 (70)	44 (30)	45 (30)
6	Szeimies et al. 2010 [24]	Germany	RCT	360	132	130	NA	NA	NA	NA	NA	NA
7	Karrer et al. 2021 [25]	Germany	RCT	55	29	26	60–85	61–84	24 (82.8)	23 (79.3)	5 (17.2)	6 (20.7)

Abbreviations: NA, not applicable; PDT, photodynamic therapy; RCT, randomized controlled trial.

**TABLE 2:** Characteristics of management.

Article ID	Author, year	Photodynamic therapy				Cryotherapy		Lesion preparation (scraping, curettage)	
		Illumination source	Mean wavelength	Photosensitizer	Occlusive dressing application	Number of cycles	Exposure duration mean ± SD	PDT	Cryotherapy
1	Szeimies et al. 2002 [20]	Red light	620 nm	MAL	Yes	2	24 ± 18 s	Yes	Yes
2	Freeman et al. 2003 [21]	Red light	620 nm	MAL	Yes	1	18 ± 19 s	Yes	No
3	Morton et al. 2006 [22]	Red light	630 nm	MAL	Yes	2	16 ± 7 s	Yes	No
4	Kaufmann et al. 2008 [19]	Red light	630 nm	MAL	Yes	2	20 ± 14 s	Yes	No
5	Hauschild et al. 2009 [23]	Red light	630 nm	ALA‐patch	No	1	7.3 ± 2 s	No	No
6	Szeimies et al. 2010 [24]	Red light	630 nm	ALA‐patch	No	1	7.3 ± 2 s	No	No
7	Karrer et al. 2021 [25]	Sunlight	NA	MAL	Yes	1	6.3 ± 1.8 s	Yes	No

Abbreviations: ALA, aminolevulinic acid; MAL, methyl aminolevulinate; NA, not applicable; nm, nanometer; PDT, photodynamic therapy.

### 3.3 A Description of Patient Characteristics of the Included Studies

Fitzpatrick skin type was reported in three studies with a total of 532 patients across both treatment modalities, with Type II being the most reported (*n* = 318, 59.77%) and Type IV the least reported (*n* = 6, 1.13%). The majority of patients treated with PDT were Type II (*n* = 152, 57.36%). Similarly, in the cryotherapy group, Type II was the most common (*n* = 166, 62.17%). Moreover, the mean lesion counts in the PDT and the cryotherapy groups for each study were 523.7 (range 208–758) and 491 (range 186–743), respectively. The mean lesion diameter ranged from 7.7 ± 5.4 to 10.3 ± 7.0 mm in the PDT group, and 7.7 ± 5.0 to 10.2 ± 5.6 mm in the cryotherapy group. Regarding reported clinical grading for AK lesions in the PDT and cryotherapy groups, 1588 (50.4%) and 1545 (50.8%) were mild or thin, respectively. PDT was applied to 1213 facial lesions (43.9%), while cryotherapy was administered to 1114 (46.6%) facial lesions [19, 20, 21, 22, 23, 24, 25].

### 3.4 Lesion Clearance

**FIGURE 2:**
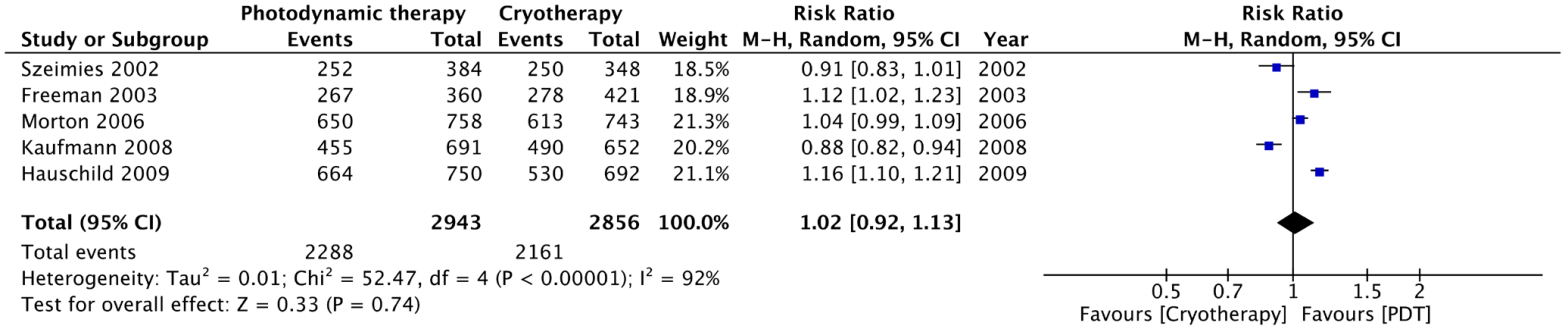
Lesion clearance forest plot. This forest plot shows the risk ratios and 95% confidence intervals (CI) for 100% lesion clearance between PDT and cryotherapy using Random‐effects model. The diamond represents the pooled effect estimate; the edges reflect the confidence interval and the height, the point estimate.

Using a pool of five studies, we assessed lesion clearance between photodynamic therapy (PDT) and cryotherapy using Random‐effects model. We found that the mean clearance rate for PDT was 79.17% (range: 63.5%–91%), while that of cryotherapy was 72.47% (range: 52.4%–88%) [19, 20, 21, 22, 23, 24, 25]. The meta‐analysis revealed no statistically significant difference between the two treatments (RR, 1.02; 95% CI, 0.92–1.13; *p* = 0.74) (Figure 2). However, the statistical heterogeneity across the included studies was high (*I* ^2^ = 92%; *p* < 0.00001).

### 3.5 Exploring the High Heterogeneity

In an attempt to examine the origin of this high heterogeneity, we performed subgroup and sensitivity analyses.

#### 3.5.1 Subgroup Analysis

**FIGURE 3:**
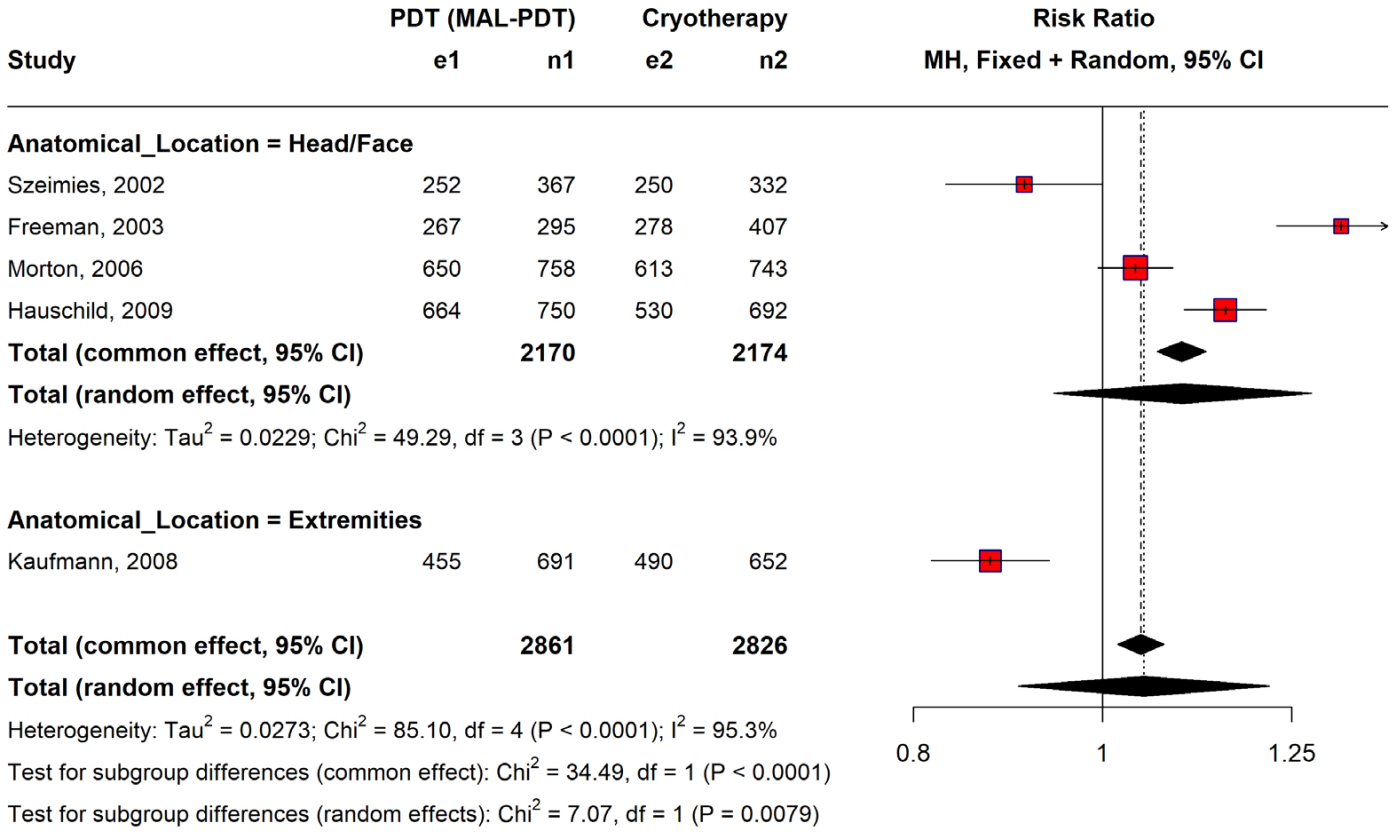
Lesion clearance forest plot with anatomical group analysis. This forest plot demonstrates the risk ratios and 95% confidence intervals for 100% lesion clearance of PDT versus cryotherapy, analyzed using a random effects model with a pre‐specified subgroup analysis by anatomical location.

We grouped the studies by body site to investigate whether the location of the lesion affected treatment efficacy. We found that efficacy differed significantly depending on the area being treated (*p*_interaction_ = 0.0079). For lesions on the head and face, PDT and cryotherapy were equally effective (4 studies; RR, 1.10; 95% CI: 0.94–1.28; *p* = 0.24). However, one study [19] found that cryotherapy was more effective for lesions on the extremities (arms and legs) (RR, 0.88; 95% CI, 0.82–0.94; *p* < 0.05) (Figure 3). Although these results suggest that lesion location may influence the treatment outcome, the findings for extremities should be interpreted with caution since they are based on one trial solely.

#### 3.5.2 Sensitivity Analysis

**FIGURE 4:**
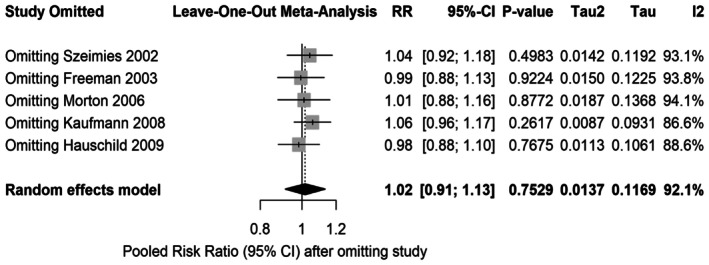
Leave‐one‐out sensitivity analysis for lesion clearance. This plot shows the pooled Risk Ratio after omitting one study at a time. The *I* ^2^ = 86.6% value obtained after omitting the Kaufmann et al. [19] study shows that this study was a major contributor to heterogeneity.

We also performed a leave‐one‐out analysis to see if any single study was responsible for the high heterogeneity (*I* ^2^ = 92%) in our results. The outcome of the analysis remained the same. There was no statistically significant difference between the two treatments (Figure 4). When we excluded the Kaufmann et al. [19] study, the heterogeneity dropped the most (to 86.6%). This suggests that the location of the skin lesions (like the arms or legs) was likely a reason why some study results differed from others.

### 3.6 Cosmetic Outcomes

**FIGURE 5:**
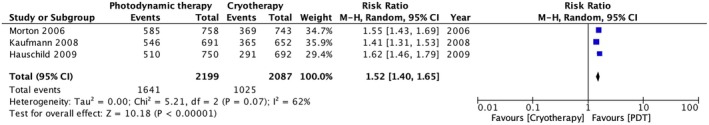
Excellent cosmetic outcomes forest plot. This forest plot shows risk ratios (RR) and 95% confidence interval (CI) for excellent cosmetic outcomes clearance of PDT versus cryotherapy using random‐effect model. The diamond represents the pooled effect estimate; the edges reflect the confidence interval and the height, the point estimate.

PDT showed excellent cosmetic outcomes with a mean of 77.82% (range: 68%–83%), while cryotherapy had fewer excellent results with a mean of 50.34% (42%–56%) [19, 20, 21, 22, 23, 24, 25]. A forest plot was conducted to compare cosmetic outcomes between patients who underwent PDT and those who received cryotherapy. The Random‐effects model indicated that PDT resulted in statistically significantly higher cosmetic outcome rates (74.62% vs. 49.11%: RR, 1.52; 95% CI, 1.4–1.65; *p* < 0.00001) [19, 22, 23]. The heterogeneity across studies was moderate (*I* ^2^ = 62%, *p* = 0.07) (Figure 5).

### 3.7 Patient Satisfaction

Patient satisfaction was higher for PDT than Cryotherapy. Data were available for 394 PDT patients (53.3%) and 259 cryotherapy patients (35.3%). 62.4% (*n* = 246) of PDT patients reported “very satisfied”, compared to 61.4% (*n* = 159) in the cryotherapy group. Similarly, 35.8% (*n* = 141) of PDT patients were “satisfied”, while only 28.6% (*n* = 74) in the cryotherapy group. This indicates higher satisfaction with PDT than with cryotherapy and suggests PDT is potentially the preferred treatment modality in terms of patient reported experience.

### 3.8 Post‐Procedure Adverse Events

The analysis of post‐procedure adverse events (AEs) revealed notable differences between PDT and cryotherapy. We focused on the specific adverse events that were clinically important and differed between the two treatments, rather than combining all events into a single total adverse events measure. The acute pain/burning profile was quantitatively assessed using data from Szeimies et al. [14]. This analysis combined the reported events of “burning sensation” and “skin pain” (42% of PDT patients vs. 22% of cryotherapy patients), demonstrating that PDT was associated with a statistically significant higher risk of burning/pain relative to cryotherapy (RR, 1.95; 95% CI, 1.27–3.02; *p* = 0.0025).

Conversely, the predominant adverse effects documented for cryotherapy were tissue damage sequelae such as vesicle or blister formation. Data from Hauschild et al. [23] imply that cryotherapy was associated with a significantly higher risk of vesicles/blisters (57.7% vs. 31.8%; RR, 0.55; 95% CI, 0.41–0.74; *p* < 0.0001). The total and specific adverse event rates across all studies are summarized in Table 3. Other notable findings included significantly higher rates of hypopigmentation following PDT (88% vs. 33% in Freeman et al.). Patient withdrawal due to procedure‐related adverse events was observed in Szeimies et al. (*n* = 1, 0.98% for PDT) and Morton et al. (*n* = 1, 0.84% for PDT), with Szeimies et al. also documenting the withdrawal of two patients (2%) because of cryotherapy‐related pain [19, 20, 21, 22, 23].

**TABLE 3:** Summary of specific and total adverse events by study.

Study	Treatment	Total AEs (any event) (*n*/*N*)	Differentiating AE 1: burning/pain (*n*/*N*)	Differentiating AE 2: vesicles/blisters (*n*/*N*)
Szeimies et al. 2002 [20]	PDT (*N* = 102)	43/102 (42.2%)	43/102 (42.2%)	NA
	Cryo (*N* = 100)	22/100 (22.0%)	22/100 (22.0%)	NA
Freeman et al. 2003 [21]	PDT (*N* = 88)	64/88 (72.7%)	NA	NA
	Cryo (*N* = 89)	31/89 (34.8%)	NA	NA
Morton et al. 2006 [22]	PDT (*N* = 119)	74/119 (62.2%)	High incidence (Narrative)	NA
	Cryo (*N* = 119)	86/119 (72.3%)	Low incidence (Narrative)	NA
Kaufmann et al. 2008 [19]	PDT (*N* = 121)	52/121 (43.0%)	High incidence (Narrative)	NA
	Cryo (*N* = 121)	75/121 (62.0%)	Low incidence (Narrative)	NA
Hauschild et al. 2009 [23]	PDT (*N* = 148)	147/148 (99.3%)	NA	47/148 (31.8%)
	Cryo (*N* = 149)	146/149 (98.0%)	NA	86/149 (57.7%)

*Note:* Reported in this specific comparative adverse event analysis. Percentages in bold indicate data points used for quantitative risk ratio analysis.

Abbreviation: NA, not available.

### 3.9 Analyzing Biases, Assessing Quality, and Determining the Level of Evidence

The risk of bias was assessed by two authors using the Cochrane Risk of Bias 2 (RoB 2) tool [18]. Both authors reached similar conclusions regarding potential biases, ensuring consistency in the evaluation. The RoB 2 assessment identified a high risk of bias in five studies due to issues in the randomization process; one study had a low risk, and one study had some concerns. Bias related to deviations from intended interventions, missing outcome data, and measurement bias was generally low, with some concerns in select trials. Notably, selective reporting bias was low across all studies (Table 4). The high risk of bias, particularly in randomization, presents a major limitation to the overall strength of evidence. The high statistical heterogeneity (*I* ^2^ > 90%) for key outcomes also reduces the reliability of the pooled estimates, lowering the certainty of the overall findings.

**TABLE 4:**
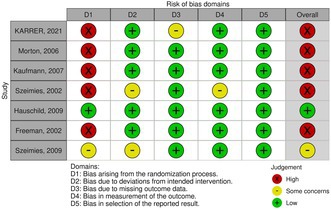
Risk of bias.

## 4 Discussion

This systematic review and meta‐analysis of seven studies aimed to compare the effectiveness of cryotherapy and PDT in treating AK. Meta‐analysis was done on lesion clearance, cosmetic outcomes, patient satisfaction, and AEs. All the studies utilized red light PDT, except Karrer et al. who employed daylight PDT, with cryotherapy as the comparator. Prior to PDT, all studies applied either MAL cream or ALA patches as photosensitizers. Three studies utilized dual cryotherapy cycles, while the other studies conducted a single cycle [19, 20, 22]. The treatment interval for the majority of the studies was 12 weeks.

Our results indicate no statistically significant difference in complete lesion clearance between PDT and cryotherapy (RR, 1.02; 95% CI, 0.92–1.13; *p* = 0.74), which is consistent with the findings of Fayter et al. [26]. Moreover, Karrer et al. also found no statistically significant difference in the efficacy of daylight‐PDT and cryotherapy, reporting complete lesion clearance rates of 63.5% (95% CI: 52.6–74.5) and 52.4% (95% CI: 41.3–63.5), respectively (*p* = 0.154) [25]. That said, Szeimies et al. [14] found cryotherapy more effective than MAL‐PDT in achieving complete lesion response. This may be attributed to the implementation of double freeze–thaw cycles with a mean cumulative freezing time of 24 s, enhancing clearance through better thermal modulation and cellular response.

The high heterogeneity (*I* ^2^ = 92%) we found in the primary outcome of lesion clearance necessitated subgroup analysis. The analysis demonstrated that the overall non‐significant finding masked a critical effect modification by anatomical location (*p*_interaction_ = 0.0079). In particular, PDT and cryotherapy showed comparable efficacy for head and face lesions (RR, 1.10; 95% CI, 0.94–1.28; *p* = 0.24). However, cryotherapy appeared better for extremity lesions (RR, 0.88; 95% CI, 0.82–0.94; *p* < 0.05) in an exploratory analysis of a single trial (Kaufmann et al.) [19]. This finding is exploratory and must be confirmed by more trials. Nevertheless, the finding is supported by previous studies done by Szeimies et al. [27] and Kurwa et al. [28], who concluded that PDT is less effective for AK lesions located on the extremities than those on the face or scalp.

We performed a leave‐one‐out sensitivity analysis (Figure 4) to identify the possible cause for the high heterogeneity of the pooled results. The analysis showed the lack of a statistically significant difference regardless of which study was omitted. However, removing Kaufmann et al. [19] reduced heterogeneity to 86.6%, proposing it as a primary outlier due to the anatomical site treated. In contrast, Morton et al. found MAL‐PDT more effective than cryotherapy at 12 weeks (84.4% vs. 74.5%) with a single session and comparable at 24 weeks (86.7% vs. 83.9%) after retreatment. These results suggest MAL‐PDT is superior in achieving complete clearance with fewer sessions, enhancing patient convenience and reducing healthcare visits.

Regarding cosmetic outcomes and patient satisfaction, the meta‐analysis showed that PDT offers superior cosmetic outcomes and a potential higher patient satisfaction (RR, 1.43; 95% CI, 0.91–2.25; *p* = 0.12). However, given the open‐label nature of the included trials, these particular findings are highly susceptible to performance and detection bias. Kaufmann et al. reported greater satisfaction with PDT, despite superior lesion clearance by cryotherapy on the extremities, suggesting that extended exposure and increased freeze cycles may enhance clearance, yet potentially result in more pronounced cosmetic sequelae and reduced patient satisfaction. Additionally, Fayter et al. and Patel et al. also reported superior cosmetic outcomes with PDT, which aligns with our conclusion [26, 29]. Gupta et al. concluded that ALA‐PDT was superior to cryotherapy; however, with respect to PDT using MAL, the authors refrained from conducting a detailed analysis due to the high heterogeneity observed within the results [30].

Safety profiles differed between PDT and cryotherapy with varying tolerability issues. The analysis established that the most significant patient discomfort associated with PDT is acute pain/burning. Based on the event percentages (42% vs. 22%) of Szeimies et al., PDT significantly increased the likelihood of this adverse event (RR, 1.95; 95% CI, 1.27–3.02; *p* = 0.002). Conversely, the predominant adverse effect documented for cryotherapy was tissue damage sequelae like vesicle or blister formation, which was significantly higher following cryotherapy (RR, 0.55; 95% CI, 0.41–0.74; *p* < 0.001). Moreover, Szeimies et al. (*n* = 1, 0.98%), and Morton et al. (*n* = 1, 0.84%), recorded patient withdrawal as a result of adverse events associated with PDT [20, 22]. These specific characteristics highlight that treatment decision‐making is a trade‐off between acute pain (PDT) and visible post‐procedure sequelae (cryotherapy) [20].

These findings have several clinical implications. They indicate that the selection between PDT and cryotherapy should be individualized based on disease severity, anatomical location, and lesion extent. Findings from our meta‐analysis suggest that PDT is preferable in cases where aesthetic outcomes are a priority, while cryotherapy may be more suitable for less visible anatomical regions given the comparable efficacy of both modalities in lesion complete clearance. Furthermore, cryotherapy might be particularly indicated for single, hyperkeratotic lesions and for patients requiring a prompter therapeutic intervention. These observations are consistent with the findings of Salman et al. who identified cryotherapy as the treatment of choice for patients with a limited number of lesions (typically 1–6 lesions) or isolated lesions [31]. The factors influencing the choice between PDT and cryotherapy were explored by Erlendsson et al. who found that cryotherapy was frequently employed in scattered solitary lesions and less commonly in cases with multiple AK lesions [32]. Nevertheless, treatment costs must also be factored into decision‐making. Cryotherapy is a cheaper, faster, and widely accessible option, whereas PDT is relatively expensive and necessitates lesion preparation and photosensitizer application.

One of the main strengths of this study is the thorough and systematic search across multiple databases, ensuring that relevant studies were identified and reducing the risk of selection bias. The application of meta‐analysis allowed for a quantitative synthesis of data across studies, providing more precise estimations of treatment effects and increasing statistical power. A key limitation of this study is the high risk of bias (five out of seven articles scored a high level of risk), which could be attributed to the flawed randomization process (selection bias) using the RoB 2 tool. The absence of blinding in all included trials, combined with the flawed randomization, significantly impacts the reliability of the evidence. Consequently, subjective outcomes such as cosmetic results and patient satisfaction are highly susceptible to performance and detection bias, potentially leading to an overestimation of the superiority of PDT. Furthermore, the high statistical heterogeneity further complicates outcome interpretation and reduces the reliability of treatment comparisons. In addition, variability in cryotherapy protocols demands careful interpretation of comparative effectiveness. Another limitation is the inconsistent use of per‐protocol analysis rather than intention‐to‐treat analysis in some studies, which may have biased treatment effectiveness estimates, modifying the applicability of the findings.

Based on above‐mentioned observations, we offer several recommendations for future research. To improve reliability and comparability, future RCTs should focus on standardized and controlled procedures, including unified guidelines for lesion preparation, treatment duration, frequency of re‐treatment, result reporting, adverse event documentation, and long‐term follow‐up. Given the open‐label nature of the included RCTs, blinded post‐treatment assessments are encouraged to enhance validity and objectivity. Furthermore, future research should investigate the cost‐effectiveness of PDT versus cryotherapy incorporating both direct costs (e.g., treatment expenses, clinician fees) and indirect costs (e.g., follow‐up visits, transportation, lost productivity) to optimize resource allocation and enhance patient accessibility.

## 5 Conclusion

In conclusion, our meta‐analysis indicates that the efficacy of photodynamic therapy (PDT) versus cryotherapy in achieving complete lesion clearance is comparable and potentially dependent on anatomical location. While both treatments are comparable for head and face lesions, Cryotherapy showed better results for extremity lesions in one trial, requiring further research to explore this finding. From a patient‐centered perspective, PDT is preferable due to better cosmetic results and higher patient satisfaction. While the superior subjective outcomes for PDT are encouraging, the high risk of bias in the included trials calls for cautious interpretation. We suggest that future research enhance the current understanding of the comparative efficacy and outcomes to include a longer follow‐up duration and to standardize the treatment protocols.

## Author Contributions

**Saleh Aldraibi:** conceptualization, data curation, formal analysis, investigation, project administration, resources, visualization, writing – original draft, writing – review and editing. **Maya Alharbi:** conceptualization, data curation, methodology, investigation, project administration, visualization, writing – original draft, writing – review and editing. **Kholoud Alzubaidi:** conceptualization, investigation, writing – original draft, writing – review and editing. **Omar Turkistani:** conceptualization, investigation, writing – original draft, writing – review and editing. **Abdulmalik Bin Kassim:** conceptualization, investigation, writing – original draft, writing – review and editing. **Dhai Almutairi:** conceptualization, investigation, writing – original draft, writing – review and editing. **Majid Alhammadi:** conceptualization, investigation, writing – original draft, writing – review and editing. **Jumanah Makhtoum:** conceptualization, investigation, writing – original draft, writing – review and editing. **Fadi Alghamdi:** conceptualization, investigation, project administration, writing – review and editing, supervision, validation.

## Funding

The authors have nothing to report.

## Ethics Statement

The authors confirm that the ethical policies of the journal, as noted on the journal's author guidelines page, have been adhered to. No ethical approval was required as this is a review article with no original research data.

## Consent

The authors have nothing to report.

## Conflicts of Interest

The authors declare no conflicts of interest.

## Data Availability

Data sharing not applicable to this article as no datasets were generated or analyzed during the current study.
