# Correction to “Comparative Efficacy of Photodynamic Therapy Versus Cryotherapy for Actinic Keratosis: A Systematic Review and Meta‐Analysis of Randomized Controlled Trials”

**DOI:** 10.1111/jocd.70916

**Published:** 2026-05-14

S.Aldraibi, M.Alharbi, K.Alzubaidi, et al., “Comparative Efficacy of Photodynamic Therapy Versus Cryotherapy for Actinic Keratosis: A Systematic Review and Meta‐Analysis of Randomized Controlled Trials,” *Journal of Cosmetic Dermatology*. 25, no. 4 (2026): e70749, 10.1111/jocd.70749.

In paragraph 2 of Section 3.8 Post‐Procedure Adverse Events, the sentence “Other notable findings included significantly higher rates of hypopigmentation following PDT (88% vs. 33% in Freeman et al.)” was incorrect.

This should have read: “Other notable findings from Hauschild et al. [23] included significantly higher rates of hypopigmentation after cryotherapy (33%). In contrast, 88% of lesions treated with PDT showed normal pigmentation, while hyperpigmentation was observed in 9%.”

We apologize for this error.

